# Prognostic Value of Serum Caspase-Cleaved Cytokeratin-18 Levels before Liver Transplantation for One-Year Survival of Patients with Hepatocellular Carcinoma

**DOI:** 10.3390/ijms17091524

**Published:** 2016-09-09

**Authors:** Leonardo Lorente, Sergio T. Rodriguez, Pablo Sanz, Antonia Pérez-Cejas, Javier Padilla, Dácil Díaz, Antonio González, María M. Martín, Alejandro Jiménez, Manuel A. Barrera

**Affiliations:** 1 Intensive Care Unit, Hospital Universitario de Canarias, Ofra, s/n. La Laguna, Tenerife 38320, Spain; 2 Intensive Care Unit, Hospital Universitario Nuestra Señora Candelaria, Crta Rosario s/n, Santa Cruz Tenerife 38010, Spain; sergiotomasr@hotmail.com (S.T.R.); mar.martinvelasco@gmail.com (M.M.M.); 3 Deparment of Surgery, Hospital Universitario Nuestra Señora de Candelaria, Crta Rosario s/n, Santa Cruz Tenerife 38010, Spain; sanzpereda@gmail.com (P.S.); javipq@me.com (J.P.); mbargom@yahoo.es (M.A.B.); 4 Laboratory Deparment, Hospital Universitario de Canarias, Ofra, s/n. La Laguna, Tenerife 38320, Spain; aperezcejas@gmail.com; 5 Deparment of Digestive, Hospital Universitario Nuestra Señora de Candelaria, Crta Rosario s/n, Santa Cruz Tenerife 38010, Spain; ddiazbet@gmail.com (D.D.); angonrod@gobiernodecanarias.org (A.G.); 6 Research Unit, Hospital Universitario de Canarias, Ofra, s/n. La Laguna, Tenerife 38320, Spain; ajimenezsosa@gmail.com

**Keywords:** cytokeratin, hepatocellular carcinoma, liver transplantation, mortality, outcome

## Abstract

Cytokeratin (CK)-18 is the major intermediate filament protein in the liver and during hepatocyte apoptosis is cleaved by the action of caspases; the resulting fragments are released into the blood as caspase-cleaved cytokeratin (CCCK)-18. Higher circulating levels of CCCK-18 have been found in patients with hepatocellular carcinoma (HCC) than in healthy controls and than in cirrhotic patients. However, it is unknown whether serum CCCK-18 levels before liver transplantation (LT) in patients with HCC could be used as a prognostic biomarker of one-year survival, and this was the objective of our study with 135 patients. At one year after LT, non-survivors showed higher serum CCCK-18 levels than survivors (*p* = 0.001). On binary logistic regression analysis, serum CCCK-18 levels >384 U/L were associated with death at one year (odds ratio = 19.801; 95% confidence interval = 5.301–73.972; *p* < 0.001) after controlling for deceased donor age. The area under the receiver operating characteristic (ROC) curve of serum CCCK-18 levels to predict death at one year was 77% (95% CI = 69%–84%; *p* < 0.001). The new finding of our study was that serum levels of CCCK-18 before LT in patients with HCC could be used as prognostic biomarker of survival.

## 1. Introduction

Hepatocellular carcinoma (HCC) is one of the most frequent malignancies, the most frequent primary liver malignancy, and the second most frequent cause of cancer-related death worldwide. Each year, 600,000 new cases of HCC are diagnosed and 750,000 patients die of HCC throughout the world. For certain patients with HCC, the treatment of choice is liver transplantation (LT), which treats the underlying liver disease and removes the primary tumor [1,2,3,4,5,6].

The apoptotic process leads to active elimination of cells by a programmed pathway. Apoptosis is increased in liver diseases [7,8,9]. Cell death by apoptosis occurs mainly by two pathways, the extrinsic and the intrinsic pathways. In the extrinsic pathway, the apoptosis is started by the activation of the tumor necrosis factor receptor superfamily (TNFRSF) by its ligand (TNFSF). In the intrinsic pathway, apoptosis is activated by interleukin (IL)-1, IL-6, oxygen free radicals and nitric oxide (NO), which leads to the liberation of cytochrome from mitochondria. Both pathways activate caspase 3 leading to cell death. Hepatocytes express surface death receptors (which initiate the extrinsic cell death pathway) and require mitochondrial amplification for the intrinsic cell death pathway [7].

Cytokeratin 18 (CK-18), the major protein in liver intermediate filament and present in most parenchymal and epithelial cells [10], may be cleaved by caspases and could be released into the blood [11], and during hepatocyte apoptosis, caspase-cleaved cytokeratin (CCCK)-18 appear in blood [7,8,9]. CCCCK-18 can be determined by a monoclonal antibody (M30) that recognizes the CK-18 Asp 396 neoepitope [12,13].

Higher circulating levels of CCCK-18 have been found in patients with different tumoral diseases than in healthy controls [14,15] and have even been associated with prognosis of different tumoral diseases [16,17,18,19,20]. In addition, higher circulating levels of CCCK-18 have been found in patients with HCC than in healthy controls [21,22], and than in patients with cirrhosis [23,24]. In addition, one study has found an association between serum CCCK-18 levels and survival in patients with HCC [25]. However, such association has not been reported in HCC patients undergoing LT; thus, the objective of the current study was to determine whether that association exists in this population.

## 2. Results

Of 135 HCC patients, 15 (11.1%) died within one year of LT. Table 1 shows the comparison of clinical and demographic characteristics between one-year survivors (*n* = 120) and non-survivors (*n* = 15). We found that one-year non-survivors showed higher LT-donor age (*p* = 0.03) and serum levels of CCCK-18 (*p* = 0.001) than survivors; however, only serum levels of CCCK-18 were significantly higher in non-survivors than in survivor patients after Bonferroni correction. In addition, we found no statistically significant differences between one-year non-surviving and surviving patients in ABO blood type, Child–Pugh score, degree of tumor differentiation, sex, infiltration, inside Milan criteria before and after LT, macrovascular invasion, microvascular invasion, multinodular tumor, portal hypertension, LT technique, pre-LT treatment, age of LT recipient, model for end-stage liver disease (MELD) score, serum AFP levels, and nodule size.

The causes of death in the 15 non-surviving patients at one year were the following: eight (53.3%) sepsis, four (26.7%) multiple organ failure, two (13.3%) HCC recurrence, and one (6.7%) recurrence of hepatitis C virus infection. 

Multiple logistic regression analysis showed that serum CCCK-18 levels >384 U/L were associated with death at one year (odds ratio = 19.801; 95% confidence interval = 5.301–73.972; *p* < 0.001) after controlling for deceased donor age (Table 2). In addition, serum CCCK-18 levels >384 U/L were also associated with death at five years in logistic regression analysis (odds ratio = 5.154; 95% confidence interval = 1.880–14.126; *p* < 0.001) after controlling for deceased donor age.

**Figure 1:**
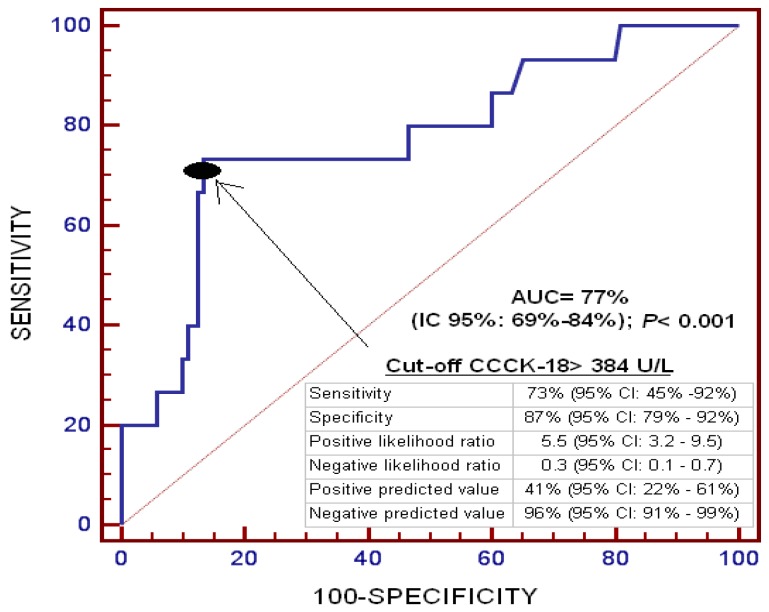
Receiver operating characteristic curve of serum caspase-cleaved cytokeratin (CCCK)-18 levels to predict mortality at one year in patients undergoing liver transplantation for hepatocellular carcinoma.

On ROC analysis, the area under the curve for serum CCCK-18 levels to predict death at one year was 77% (95% CI = 69%–84%; *p* < 0.001) (Figure 1).

**Figure 2:**
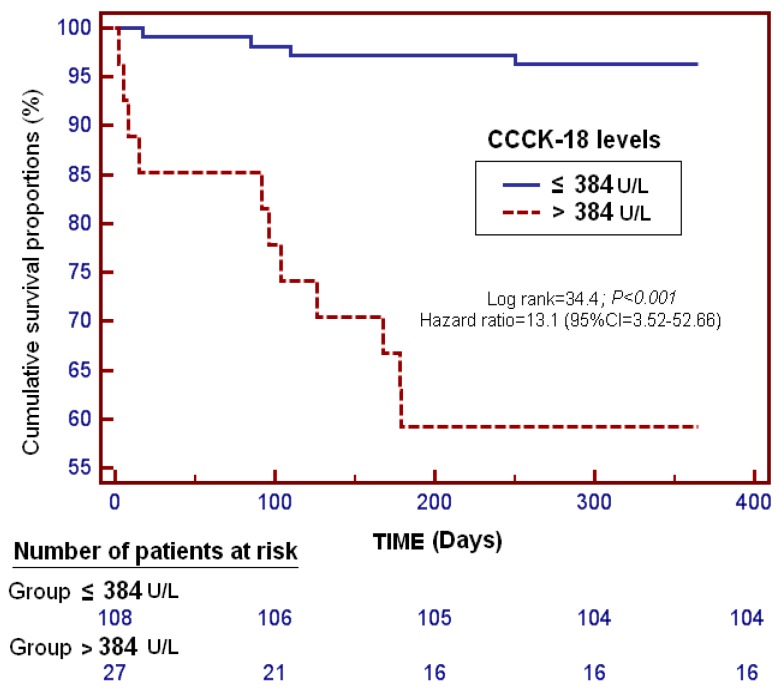
Survival curves at one year using serum caspase-cleaved cytokeratin (CCCK)-18 levels higher or lower than 384 U/L in patients undergoing liver transplantation for hepatocellular carcinoma.

Kaplan–Meier survival analysis showed that patients with serum CCCK-18 levels higher than 384 U/L had a higher risk of death at one year (Hazard Ratio = 13.1 (95% CI = 3.52–52.66); *p* < 0.001) than those with lower levels (Figure 2).

We found an association between serum levels of CCCK-18 and AFP (rho = 0.28; *p* = 0.001). However, we found no statistically significant differences in serum CCCK-18 levels according to ABO blood type (*p* = 0.65), Child–Pugh score (*p* = 0.87), degree of tumor differentiation (*p* = 0.90), sex (*p* = 0.25), infiltration (*p* = 0.33), inside Milan criteria before LT (*p* = 0.35), inside Milan criteria after LT (*p* = 0.24), macrovascular invasion (*p* = 0.99), microvascular invasion (*p* = 0.79), multinodular tumor (*p* = 0.33), portal hypertension (*p* = 0.13), LT technique (*p* = 0.89) and pre-LT treatment (*p* = 0.44). Neither did we find an association between serum CCCK-18 levels and LT recipient age (*p* = 0.27), MELD score (*p* = 0.06) and nodule size (*p* = 0.84). Nor did we find an association between serum CCCK-18 levels and leukocytes count (*p* = 0.33), serum protein concentration (*p* = 0.52) and serum albumin concentration (*p* = 0.08). We found higher serum CCCK-18 levels before LT in patients that developed multiple organ failure after LT (*p* < 0.001) but not in patients that developed sepsis after LT (*p* = 0.09).

We found statistically significant differences in the distribution of causes of death between patients with higher and lower serum CCCK-18 levels than 384 U/L (*p* = 0.02). The causes of death in the 11 patients with serum CCCK-18 levels higher than 384 U/L were: seven (63.6%) sepsis and four (36.4%) multiple organ failure. In addition, the causes of multiple organ failure were in two cases LT primary non-function and in two cases massive bleeding. The causes of death in the four patients with serum CCCK-18 levels lower than 384 U/L were: two (50.0%) HCC recurrence, one (25.0%) recurrence of hepatitis C virus infection, and one (25.0%) sepsis. 

## 3. Discussion

To our knowledge, this is the first study to report circulating levels of serum CCCK-18 in patients with HCC before undergoing LT. The new findings of our study were that survivors at one year after LT for HCC showed lower serum CCCK-18 levels before LT than non-surviving patients, that there was an association between serum CCCK-18 levels before LT and one-year survival after LT, controlling for deceased donor age, and that pre-LT serum levels of CCCK-18 in patients with HCC could be used as a prognostic biomarker of survival.

We found a survival rate of 88.9% at one year of LT for HCC, which falls within the range of previous publications (75%–95%) [26,27,28,29,30,31,32,33].

Our findings are in accordance with those of previous studies showing that circulating CCCK-18 levels are associated with the prognosis of patients with different tumoral diseases [16,17,18,19,20], and also of patients with HCC [25]. In addition, circulating CCCK-18 levels have been associated with the prognosis of patients with other processes such as sepsis [34] and brain trauma injury [35]. Higher circulating levels of CCCK-18 have been previously reported in patients with HCC than in healthy controls [21,22], and in patients with cirrhosis [23,24]; however, our study is the first to report an association between serum CCCK-18 levels before LT and one-year survival after LT for HCC, and that serum levels of CCCK-18 before LT in patients with HCC could be used as a prognostic biomarker of survival. In a previous study by Brenner et al. with 100 patients undergoing LT (28.6% HCC, 19.5% ethyl-toxic cirrhosis, 7.8% viral hepatitis, and 44.1 other causes), an association between circulating CCCK-18 levels before transplantation and prognosis was not found [36]. 

Different factors have been associated with worse prognosis in patients with HCC undergoing LT, such as serum AFP levels, tumor number, tumor size, degree of differentiation, macro- and microvascular hepatic invasion, outside Milan criteria and infiltration [4,37,38]. However, in our study, only differences in liver donor age were found (showing lower donor age in one-year survivors than non-survivors).

Previous studies have found that circulating CCCK-18 levels were associated with the presence of metastasis [17], serum levels of AFP [18] and tumor size [19,20]. In the present study, we found that serum levels of CCCK-18 were associated with AFP but not with other variables such as sex, age, Child–Pugh score, MELD score, degree of tumor differentiation, infiltration, Milan criteria, macro- and microvascular invasion, multinodular tumor, nodule size or portal hypertension.

The most frequent causes of death vary considerably in previously published studies on HCC patients [26,27,28,29,30,31], and they include recurrence of HCC, recurrence of hepatitis C virus infection. sepsis, and multiple organ failure. In addition, the causes of death in our study were sepsis (53.3%), multiple organ failure (26.7%), HCC recurrence (13.3%), and recurrence of hepatitis C virus infection (6.7%). 

Certain limitations in our study should be recognized. First, other potentially confounding factors not related to HCC (e.g., hepatitis viral infection, diet, stability in serum) could have affected the determination of serum concentrations of CCCK-18 levels; Second, we did not measure serum levels of CCCK-18 in healthy subjects and non-HCC cirrhotic patients; however, the aim of our study was to determine whether there is an association between serum CCCK-18 levels and survival in patients with HCC undergoing LT, and not to compare serum levels of CCCK-18 between HCC cirrhotic patients, non-HCC cirrhotic patients and healthy subjects; Third, we did not measure other compounds of apoptosis; Fourth, we did not record data on apoptosis in liver tissue; thus, we did not determine whether there is an association between serum CCCK-18 levels and liver apoptosis; and one study using immunofluorescence staining and microscopic examination has found that cytokeratin-18 expression was significantly higher in six of the seven HCC cell lines examined than in the control cells [39]; Fifth, serum samples were not taken during patient follow-up, and so serum levels of CCCK-18 were not determined in this period. According to the findings of our study, we believe there is an association between serum CCCK-18 levels before LT for HC and one-year survival after LT; however, we can not conclude that the increase of serum CCCK-18 levels is the cause of death. It is possible that non-surviving patients maintain high serum levels of CCCK-18 and those levels decrease in survivors.

The reason by which the levels of serum CCCK-18 before LT could be associated with the prognosis of LT is uncertain. We have not found higher serum CCCK-18 levels before LT in patients that developed sepsis after LT. In addition, serum CCCK-18 levels before LT were not linked to sepsis before LT due to the fact that no patient was septic at the moment of LT, and we did not find an association between serum CCCK-18 levels and leukocyte count before LT. Neither did we find an association between serum CCCK-18 levels and pre-operative nutrient condition (assessed by serum concentrations of protein and albumin). However, we found higher serum CCCK-18 levels before LT in patients that developed multiple organ failure after LT. In addition, we have found a different distribution of causes of death according to serum CCCK-18 levels. We found that all patients with serum CCCK-18 levels higher than 384 U/L died due to sepsis (63.6%) or multiple organ failure (36.4%); however, the patients with serum CCCK-18 levels lower than 384 U/L died due to HCC recurrence (50.0%), recurrence of hepatitis C virus infection (50.0%) or sepsis (25.0%). Thus, it is possible that patients with a higher basal level of apoptosis (with higher circulating CCCK-18 levels) could have a higher risk of multiple organ failure after LT. With respect to this hypothesis, some mutations have been found in genes of proteins that modulate apoptosis [40,41,42]. In a study with Tatars from Bashkortostann, an association was found between longevity and several polymorphism, such as caspase-8 gene (*rs3834129*), 140016C>T polymorphism of Bcl2 gene (*rs12454712*) and 919A>G polymorphism of Bax gene (*rs1805419*) [40]. In one study, an association was found between polymorphism of Bax G(-248)A and lung cancer risk in Chinese population [41]. In another study, an association between the Keratin 18 R89C mutation, liver apoptosis and lethality in mice was found [42].

Despiste these limitations, we believe that the interesting points of our study are that we report for the first time that survivors at one year after LT for HCC showed lower serum CCCK-18 levels before LT than non-survivor patients, and that serum levels of CCCK-18 before LT in patients with HCC could be used as a prognostic biomarker of survival. In addition, we think that further studies are needed to corroborate these novel findings.

## 4. Materials and Methods

### 4.1. Design and Subjects

This observational, retrospective, single-center study included 135 patients with HCC treated with orthotopic LT from January 1996 to February 2015 at Hospital Universitario Nuestra Señora de Candelaria (Santa Cruz de Tenerife, Spain). All livers were from brain death donors. The study was approved by the Institutional Review Board. Written informed consent was obtained from the patients or from their family members.

### 4.2. Variables Recorded

ABO blood type, Child–Pugh score [43], degree of tumor differentiation, sex, infiltration, inside Milan criteria [44] before and after LT, macrovascular invasion, microvascular invasion, multinodular tumor, portal hypertension (determined clinically or by hepatic venous pressure gradient), LT technique, pre-LT treatment, age of liver donor, age of liver recipient, model for end-stage liver disease (MELD) score [45] by hepatic function, serum alpha-fetoprotein (AFP) levels, serum CCCK-18 levels and nodule size were recorded for each patient.

### 4.3. End-Point

Survival at one year after LT was the end-point of the study.

### 4.4. Serum CCCK-18 Analysis

Serum blood samples were collected approximately 2 h before LT. Serum levels of CCCK-18 were measured using the kit M30 Apoptosense^®^ ELISA, PEVIVA AB (Bromma, Sweden). The intra- and inter-assay coefficients of variation (CV) were <10%. The detection limit was 25 U/L. CCCK-18 levels were determined by laboratory technicians blinded to clinical data, in the Laboratory Department of the Hospital Universitario de Canarias (La Laguna, Tenerife, Spain).

### 4.5. Statistical Methods

Categorical variables are reported as frequencies and percentages. A chi-square test was used to compare categorical variables between surviving and non-surviving patient groups at one year. Continuous variables are reported as medians and interquartile ranges, and the Mann–Whitney test was used for the comparisons of continuous variables between groups.

A receiver operating characteristic (ROC) curve was plotted using serum levels of CCCK-18 as the prognostic variable and survival at one year as the classification variable. We used the Youden J index to select the optimal prognostic serum CCCK-18 cut-off value. Survival analysis was performed with a Kaplan–Meier curve using survival at one year as the dependent variable, and serum levels of CCCK-18 lower/higher than 384 U/L as the independent variable. 

Binary logistic regression analysis was used to determine the independent contribution of serum levels of CCCL-18 >384 U/L for the prediction of death during the first year of LT, controlling for age of the LT deceased donor. To estimate the clinical impact of the predictor variables, odds ratios and 95% confidence intervals were calculated. We used a Spearman’s rank correlation coefficient to determine the association between continuous variables. Differences with a *p*-value less than 0.05 were considered statistically significant. The Bonferroni method (p/k) was used to correct for multiple comparisons, where k represents the number of comparisons [46,47,48,49]. We performed statistical analyses using SPSS 17.0 (SPSS Inc., Chicago, IL, USA) and MedCal 15.2.1 (Ostend, Belgium).

## 5. Conclusions

The new finding of our study was that serum levels of CCCK-18 before LT in patients with HCC could be used as a prognostic biomarker of survival.

## Figures and Tables

**Table 1:** Clinical and demographic characteristics of surviving and non-surviving patients at one year of liver transplantation (LT) for hepatocellular carcinoma.

Clinical and Demographic Characteristics	One-Year Non-Survivors (*n* = 15)	1-Year Survivors (*n* = 120)	*p*
ABO blood type—*n* (%)			0.94
A	6 (40.0)	57 (47.5)	
B	2 (13.3)	12 (10.0)	
O	6 (40.0)	45 (37.5)	
AB	1 (6.7)	6 (5.0)	
Child–Pugh score—*n* (%)			0.37
A	10 (66.7)	57 (47.5)	
B	3 (20.0)	36 (30.0)	
C	2 (13.3)	27 (22.5)	
Degree of tumor differentiation—*n* (%)			0.53
Well-differentiated	12 (80.0)	91 (75.8)	
Moderately differentiated	2 (13.3)	26 (21.7)	
Poorly differentiated	1 (6.7)	3 (2.5)	
Female gender—*n* (%)	0	21 (17.5)	0.13
Infiltration—*n* (%)	4 (26.7)	39 (32.5)	0.77
Inside Milan criteria before LT—*n* (%)	14 (93.3)	115 (95.8)	0.51
Inside Milan criteria after LT—*n* (%)	11 (73.3)	101 (84.2)	0.29
Macrovascular invasion—*n* (%)	0	7 (5.8)	0.99
Microvascular invasion—*n* (%)	3 (20.0)	26 (21.7)	0.99
Multinodular tumor—*n* (%)	5 (33.3)	37 (30.8)	0.99
Portal hypertension—*n* (%)	11 (73.3)	81 (67.5)	0.77
Transplantation technique—*n* (%)			0.99
By-pass	6 (40.0)	45 (37.5)	
Piggy back	9 (60.0)	75 (62.5)	
Treatment for HCC before LT—*n* (%)	10 (66.7)	64 (53.3)	0.41
Percutaneous ethanol injection (PEI)—*n* (%)	7 (46.7)	28 (23.3)	0.06
Radiofrequency ablation (RFA)—*n* (%)	0	7 (5.8)	0.99
Transarterial chemoembolization (TACE)—*n* (%)	3 (20.0)	23 (19.2)	0.99
Liver resection—*n* (%)	0	3 (2.5)	0.99
Mixed treatment—*n* (%)	0	3 (2.5)	0.99
Age of liver donor (years)—median (P_25_–P_75_)	62 (49–72)	52 (35–63)	0.03
Age of LT recipient (years)—median (P_25_–P_75_)	56 (53–62)	58 (52–62)	0.96
MELD score—median (P_25_–P_75_)	15 (15–18)	15 (11–18)	0.62
Serum AFP (ng/dL)—median (P_25_–P_75_)	12.0 (4.8–164.9)	7.0 (3.8–26.4)	0.34
Serum CCCK-18 (U/L)—median (P_25_–P_75_)	401 (268–575)	254 (177–325)	1
Nodule size (cm)—median (P_25_–P_75_)	3.2 (1.7–4.6)	3.0 (2.0–3.5)	0.74
Leukocyte count—median × 10^3^/mm^3^ (P_25_–P_75_)	4.94 (3.49–7.92)	4.85 (3.59–6.18)	0.63
Total protein (g/dL)—median (P_25_–P_75_)	6.70 (5.70–7.68)	6.70 (6.05–7.10)	0.88
Albumin (g/dL)—median (P_25_–P_75_)	3.31 (2.93–4.16)	3.32 (2.91–4.05)	0.94

HCC = hepatocellular carcinoma; MELD = model for end-stage liver disease; AFP = alpha-fetoprotein; CCCK = caspase-cleaved cytokeratin; P_25_–P_75_ are percentile 25 and 75.

**Table 2:** Multiple logistic regression analysis for one-year mortality prediction in patients undergoing liver transplantation for hepatocellular carcinoma.

Predictors	Odds Ratio	95% Confidence Interval	*p*
Serum caspase-cleaved cytokeratin-18 levels >384 U/L	19.801	5.301–73.972	0.001
Age of liver donor (years)	1.048	1.002–1.096	0.04

## Abbreviations

AFP	alpha-fetoprotein
AUC	area under the curve
CCCK	caspase-cleaved cytokeratin
HCC	hepatocellular carcinoma
LT	liver transplantation
MELD	model for end-stage liver disease
